# The impact of antibiotic selection and interval time among advanced non‐small cell lung cancer patients receiving prior antibacterial treatment and first‐line chemotherapy

**DOI:** 10.1002/cam4.4815

**Published:** 2022-05-11

**Authors:** Xiaoman Tian, Ting Mei, Min Yu, Yanying Li, Rui Ao, Youling Gong

**Affiliations:** ^1^ Department of Thoracic Oncology, Cancer Center and State Key Laboratory of Biotherapy West China Hospital, Sichuan University Chengdu PR.China; ^2^ Department of Oncology Chengdu Jinniu District People's Hospital Chengdu PR.China; ^3^ Department of Oncology Sichuan Provincial People's Hospital Chengdu PR.China

**Keywords:** antibiotic treatment, chemotherapy, interval time, non‐small cell lung cancer, prognosis

## Abstract

**Background:**

To determine whether antibiotic use before chemotherapy is associated with chemotherapy responses and patient outcomes among NSCLC patients and define the optimal interval between chemotherapy initiation and antibiotic treatment.

**Materials and methods:**

One thousand four hundred and four advanced NSCLC patients receiving first‐line platinum‐based doublets therapy were retrospectively analyzed. Kaplan–Meier curve evaluated the impact of antibiotic use and type of antibiotics on the survival of patients. The factors affect the patient's prognosis were further confirmed by Cox regression. The optimal interval between antibiotic treatment and the initiation of chemotherapy was determined by the X‐tile program.

**Results:**

NSCLC patients of 33.5% advanced underwent broad‐spectrum antibiotic treatment prior to chemotherapy. In the chemotherapy only (Chemo) and chemotherapy plus antiangiogenesis (Chemo‐angio) treatment groups, prior antibiotic treatment was associated with worse OS (Chemo: 13.8 vs. 17.6 months, *p* < 0.001; Chemo‐angio:11.9 vs. 18.1 months, *p* = 0.012) and PFS (Chemo: 3.7 vs. 5.8 months, *p* < 0.001; Chemo‐angio: 3.1 vs. 5.9 months, *p* < 0.001). Cox regression analysis revealed prior antibiotic administration as an independent predictor of OS and PFS (HR for PFS/OS: 1.925/1.452, both *p* < 0.001). Antibiotic usage duration (HR for PFS/OS: 1.030/1.036, *p* = 0.009/0.001) and type (PFS/OS: *p* < 0.001/*p* = 0.01) also showed significant association with patient prognosis, with calculated interval time cutoff values of 2, 4, and 2 days for fluoroquinolones, *β*‐lactamase inhibitors, and cephalosporins, respectively.

**Conclusion:**

Antibiotic use before first‐line chemotherapy was associated with poor results in advanced NSCLC patients; treatment length and type being strongly correlated with patient outcomes. Appropriate prolongation of the time between two treatments may enhance patient survival. Further prospective research is however necessary.

## Graphical abstract

In clinical settings, many NSCLC patients undergo antibiotic treatment prior to the initiation of chemotherapy. Previous studies have reported that antibiotic use can affect the efficacy of immunotherapeutic treatment regimens. Whether antibiotics similarly impact chemotherapy efficacy, however, remains to be established. This study compared the effects of different antibiotic treatments on patient prognosis and to define the optimal interval between Abx treatment and chemotherapy initiation.

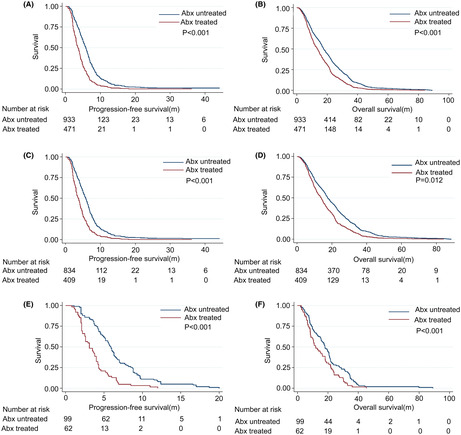

## 1 INTRODUCTION

Lung cancer is among the most common causes of cancer and cancer‐associated mortality, with approximately 85% of cases being of the non‐small cell lung cancer (NSCLC) subtype, with many patients having advanced‐stage disease at the time of initial diagnosis.^1^ In the recent years, although targeted therapy and immune checkpoint inhibitor therapy are the standard first‐line treatment options for patients with positive driver genes and positive PD‐L1 expression, respectively, platinum‐based chemotherapy remains an important treatment strategy for some patients.^2^

Advanced lung cancer patients commonly present with other respiratory complications including obstructive atelectasis, pulmonary arteriosclerosis, reduced lung elastic retraction, and respiratory mucosal epithelial fibrosis, all of which can contribute to sputum accumulation within the lung and consequent infection. As such, many patients undergo antibiotic treatment prior to the initiation of chemotherapy.

Antibiotic administration can impact gut microbiota composition.^3^ A limited number of preclinical studies have also concluded that gut microbes can impact the relative toxicity and efficacy of different anticancer treatments including chemotherapy, radiotherapy, and immunotherapy.^4^, ^5^, ^6^, ^7^

Multiple prospective and retrospective clinical studies have provided evidence that prior antibiotic therapy may impact immunotherapeutic efficacy in advanced NSCLC patients.^8^, ^9^, ^10^ Another study assessed the effects of antibiotic treatment on patients with locally advanced head and neck carcinoma undergoing chemotherapeutic and radiotherapeutic treatment, revealing that antibiotic administration negatively impacted prognostic outcomes among these patients.^11^ With respect to the impact of antibiotic treatment on chemotherapeutic efficacy, while prior studies have yielded negative results, they nonetheless observed worse overall survival (OS) and progression‐free survival (PFS) among patients that underwent antibiotic treatment.^10^, ^12^ The interval between antibiotic treatment and immunotherapy has also been found to be associated with patient prognosis.^13^, ^14^ The correlative relationship between antibiotic treatment and chemotherapy, however, remains poorly defined.

In this study, we sought to more fully clarify how prior antibiotic treatment can impact the prognosis of patients with advanced NSCLC undergoing first‐line chemotherapy, and to more fully explore the optimal interval between antibiotic administration and chemotherapy.

## 2 MATERIALS AND METHODS

### 2.1 Patients

In total, 9000 consecutive NSCLC patients treated from January 2010 to July 2018 at West China Hospital (Sichuan University), Sichuan Provincial People's Hospital, and Chengdu Jinniu District People's Hospital were retrospectively assessed for potential inclusion in this study. Individuals eligible for inclusion were patients that: (1) were diagnosed with stage IIIc or IV NSCLC without a definitive treatment option, (2) underwent first‐line platinum‐based chemotherapy with or without antiangiogenesis therapy, (3) exhibited an Eastern Cooperative Oncology Group (ECOG) performance status (PS) of 0–1, and (4) did or did not receive broad‐spectrum antibiotic treatment within 1 month prior to chemotherapy. Patients were excluded from this study if they had undergone lung surgery, exhibited positive driver gene mutation status, or underwent first‐line immunotherapeutic treatment. The antibiotic prescription and other medical characteristics were extracted from the hospital information system (HIS) in participating hospitals, as we described in our previous research.^15^

This study was approved by the ethics review committee of West China Hospital, Sichuan University, which waived informed consent.

### 2.2 Study variables

Parameters assessed in this study included age, gender, N stage, T stage, PS score, histology, hemoglobin, albumin, liver/ brain metastasis, chemotherapeutic regimen, and antibiotic (Abx) treatment. Those patients that underwent Abx treatment within 1 month prior to the initiation of first‐line platinum‐based chemotherapy were defined as the Abx‐treated group, and the others were defined as the Abx‐untreated group. The interval between the end of antibiotic treatment and the initiation of chemotherapy was additionally assessed. A duration of 7 days of antibiotic use was considered the standard course of antibiotic treatment, and more than 7 days was defined as prolonged antibiotic treatment.

### 2.3 Endpoint definition

PFS was defined as the duration of time between chemotherapy initiation and death or disease progression, while OS was defined as the time between treatment initiation and all‐cause death or most recent follow‐up. OS and PFS were the primary and secondary endpoints for this study, respectively.

### 2.4 Statistical analysis

Baseline characteristics were compared between groups via chi‐squared tests. Propensity score matching (PSM) was used to balance baseline characteristics of antibiotic‐treated and antibiotic‐untreated groups. Kaplan–Meier curves and the log‐rank test were used to compare patient PFS and OS. Univariate and multivariate Cox proportional hazards regression analyses were used to calculate hazard ratios (HRs) with 95% confidence intervals (CIs). Optimal interval duration cutoff values were calculated using the X‐tile program.^16^ The *p* < 0.05 was the threshold of significance.

### 2.5 Construction of X‐tile plots

X‐tile groups by different values as cutoffs for the target variable (low subset, high subset) and performs a statistical test on all possible groupings of the target variable. The program can choose the best split point for the data by choosing the highest χ2 value and use the log‐rank test to calculate the minimum p‐value. The color of each pixel represents the strength of the association of each cut point with survival, with red indicating a negative correlation with survival and green indicating a direct association. The horizontal x‐axis and vertical y‐axis represent all potential cut points for the low and high subsets, respectively.^16^ The best cut point occurs on the brightest pixel (red or green) identified by the software, highlighted by a black circle. Based on this cut point, the histogram will display the corresponding population, and the Kaplan–Meier survival curve will calculate the results of the survival analysis for the two groups.

### 2.6 Nomogram construction and validation

The factor of *p* < 0.05 in multivariate analysis was further used to construct the nomogram model, and the predictive power of the model was assessed using calibration curve, ROC curve, risk stratification system, and decision curve analysis (DCA).

## 3 RESULTS

### 3.1 Patient characteristics

**FIGURE 1:**
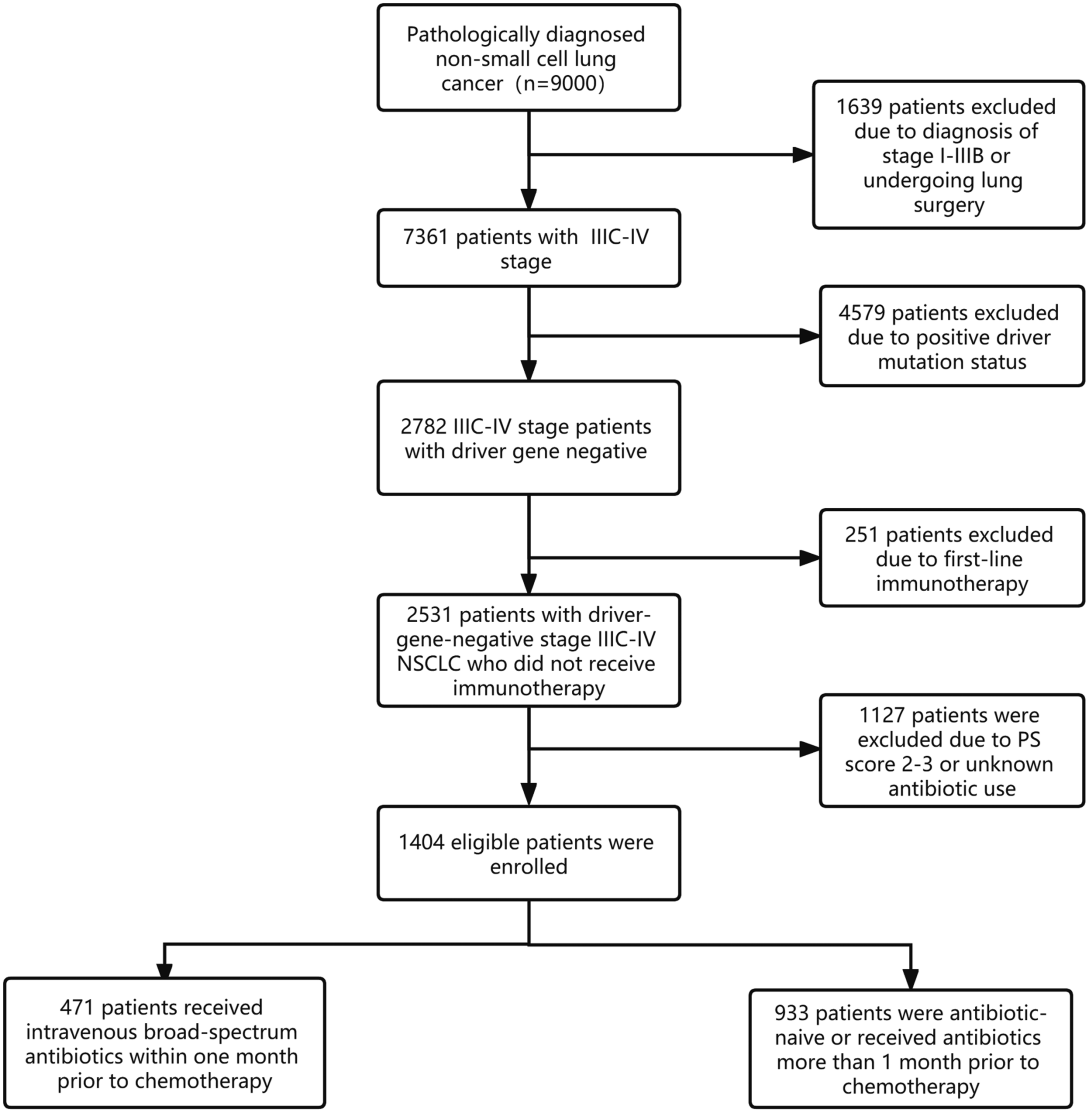
The research flow chart

In total, 1404 patients were included in the present study, of whom 471 and 933 were included in the Abx‐treated and untreated groups, respectively. The research flowchart is shown in Figure 1 and patient characteristics are compiled in Table 1. Age and brain metastases were unbalanced between antibiotic‐treated and antibiotic‐untreated groups before propensity score matching. After 1:1 matching, the baseline characteristics of the two groups were well balanced. The most commonly utilized antibiotics in the Abx group were cephalosporins [165 (33.3%), first‐ and second‐generation cephalosporins: 108, third‐ and fourth‐generation cephalosporins: 57], fluoroquinolones [157 (31.6%)], and *β*‐lactamase inhibitors [149 (35.1%), Piperacillin‐tazobactam: 115, Piperacillin‐ sulbactam: 34].

**TABLE 1:** Baseline characteristics of patients with advanced NSCLC treated with Chemotherapy

Variable	Before PSM			After PSM		
	Abx(*n* = 471)	Non‐Abx(*n* = 933)	*p*‐value	Abx(*n* = 471)	Non‐Abx(*n* = 471)	*p*‐value
Age, years						
<60	204 (43.3)	472 (50.5)	0.01	204 (43.3)	201 (42.7)	0.843
≥60	267 (56.7)	461 (49.5)		267 (56.7)	270 (57.3)	
Gender						
Male	331 (70.2)	689 (73.8)	0.156	331 (70.3)	338 (71.8)	0.615
Female	140 (29.8)	244 (26.2)		140 (29.8)	244 (26.2)	
T stage						
1	32 (6.8)	78 (8.4)	0.501	32 (6.8)	35 (7.4)	0.935
2	127 (27)	240 (25.7)		127 (27)	121 (25.7)	
3	88 (18.7)	153 (16.4)		88 (18.7)	93 (19.7)	
4	224 (47.6)	462 (49.5)		224 (47.6)	222 (47.1)	
N stage						
0	25 (5.3)	69 (7.4)	0.279	25 (5.3)	26(5.5)	0.383
1	47 (10)	73 (7.8)		47 (10)	33 (7)	
2	160 (34)	325 (34.8)		160 (34)	174 (36.9)	
3	239 (50.7)	466 (49.9)		239 (50.7)	238 (50.5)	
Histology						
Squamous	131 (27.8)	253 (27.1)	0.705	131 (27.8)	100 (21.2)	0.328
Non‐squamous	340 (72.2)	680 (72.9)		340 (72.2)	371 (78.8)	
Hemoglobin (g/L)						
≤120	163 (34.6)	319 (34.2)	0.877	163 (34.6)	152 (32.3)	0.447
>120	308 (65.4)	614 (65.8)		308 (65.4)	319 (67.7)	
Albumin (g/L)						
≤40	131 (27.8)	243 (26)	0.479	131 (27.8)	125 (26.5)	0.660
>40	340 (72.2)	690 (74)		340 (72.2)	346 (73.5)	
Liver metastasis						
Yes	67 (14.2)	125 (13.4)	0.971	67 (14.2)	93 (19.7)	0.572
No	404 (85.8)	808 (86.6)		404 (85.8)	378 (80.3)	
Brain metastasis						
Yes	72 (15.3)	205 (22.0)	0.019	72 (15.3)	90 (19.1)	0.868
No	399 (84.7)	728 (78.0)		399 (84.7)	381 (80.9)	
Chemotherapy regimen						
Platinum + pemetrexed	188 (39.9)	424 (45.4)	0.066	188 (39.9)	200 (42.5)	0.681
Platinum + Docetaxel	6 (1.3)	16 (1.7)		6 (5.5)	5 (5.5)	
Platinum + Others	12 (2.5)	40 (4.3)		12 (2.5)	18 (3.8)	
Platinum + Gemcitabine	96 (20.4)	161 (17.3)		96 (20.4)	87 (18.5)	
Platinum + Paclitaxel	169 (35.9)	292 (31.3)		169 (35.9)	161 (35.9)	
Antiangiogenic therapies						
No	409 (86.8)	834 (89.4)	0.156	409 (86.8)	406 (86.2)	0.775
Yes	62 (13.2)	99 (10.6)		62 (13.2)	65 (13.8)	

### 3.2 Survival outcomes

**FIGURE 2:**
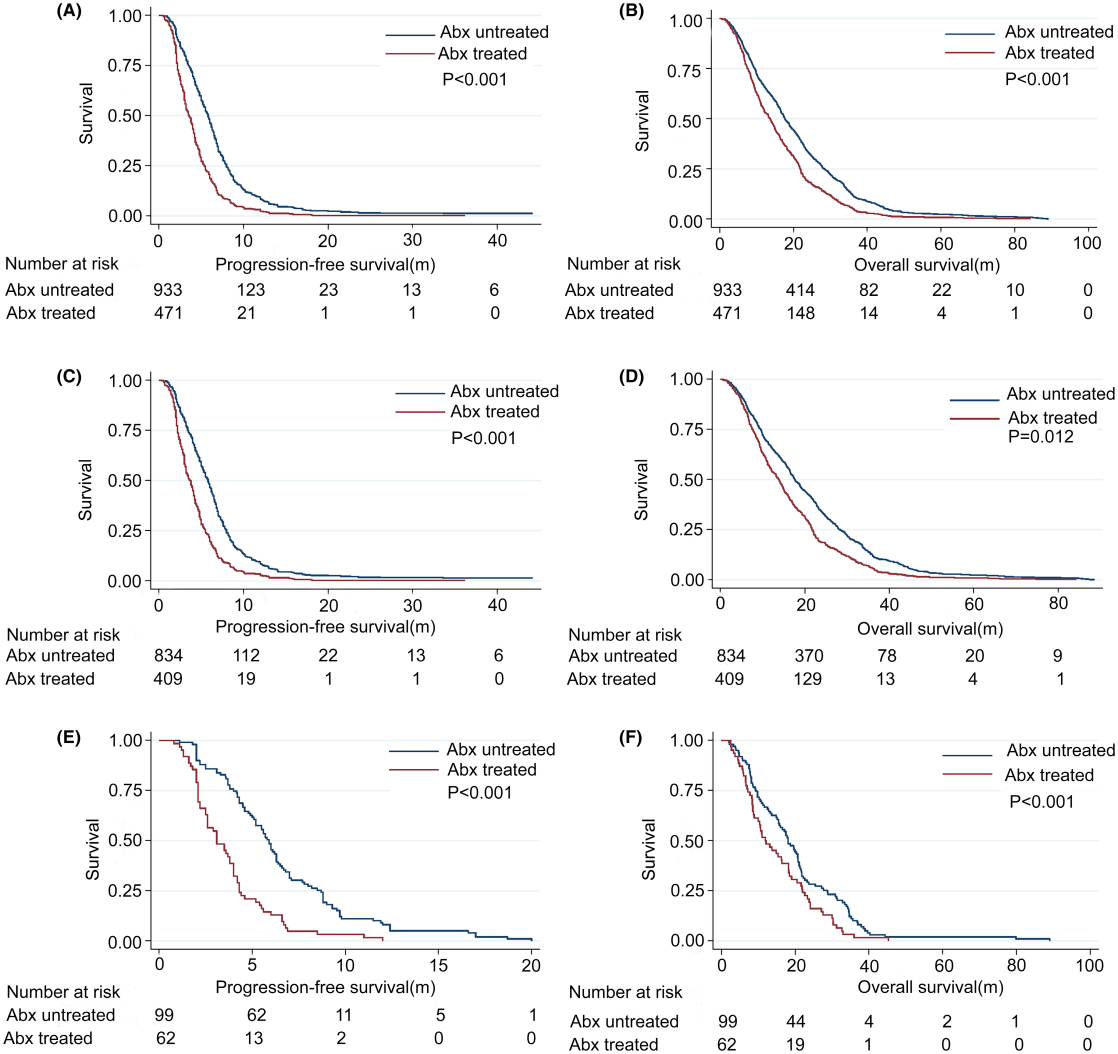
Kaplan–Meier curves for survival. (A, B) The effect of antibiotics on patients' PFS and OS in the entire population, (C, D) The effect of antibiotics on PFS and OS in patients receiving chemotherapy alone, (E, F). The effect of antibiotics on PFS and OS in patients receiving chemotherapy plus antiangiogenesis therapy

In the overall patient population, the PFS and OS of patients in the Abx group were significantly lower than those of patients in the untreated group {median PFS: 3.6 vs. 5.8 months, *p* < 0.001; median OS: 13.7 vs. 17.7 months, *p* < 0.001) (Figure 2A,B). Of patients that only underwent chemotherapeutic treatment, both PFS (median 3.7 months vs. 5.8 months, *p* < 0.001) and OS (median 13.8 months vs. 17.6 months, *p* < 0.001) were significantly reduced in the Abx treatment group relative to the untreated group (Figure 2C,D). We additionally found that among patients undergoing Chemo‐angio treatment, Abx administration was associated with significant reductions in PFS (median 3.1 months vs. 5.9 months, *p* < 0.001) and OS (median 11.9 months vs. 18.1 months, *p* = 0.012) (Figure 2E,F).

**FIGURE 3:**
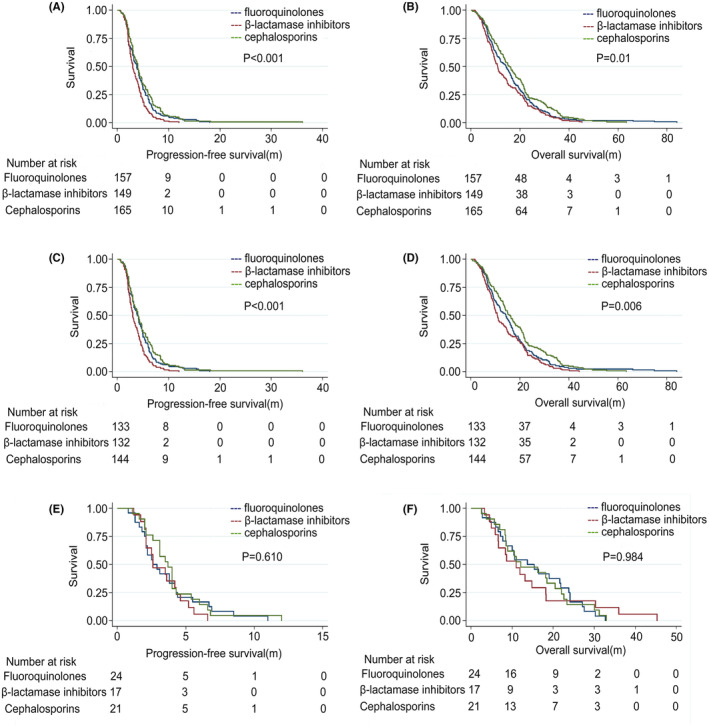
Kaplan–Meier curves of different types of antibiotics for survival. (A, B)The entire population, (C, D) Patients receiving chemotherapy alone, (E, F) Patients receiving chemotherapy plus antiangiogenesis therapy

We additionally assessed the effects of different antibiotic treatments on patient survival outcomes. Both the PFS and OS of patients that were treated with β‐lactamase inhibitors (median PFS: 3.0 months and median OS: 10.9 months) were worse than those of any other patient subgroups, followed by outcomes for patients treated with cephalosporins (median PFS: 4.0 months and median OS: 15.7 months) and fluoroquinolones (median PFS: 3.8 months and median OS: 14.3 months), respectively (PFS: *p* < 0.001 and OS: *p* = 0.001) (Figure 3A,B). The prognosis of patients in the Chemo group that underwent β‐lactamase inhibitor treatment was worse than that observed for patients in the other two Abx treatment groups (PFS: *p* < 0.001 and OS: *p* = 0.006; Figure 3C,D). In the Chemo‐angio group, the prognosis of patients receiving β‐lactam inhibitors was worse than in other groups, although the differences were not significant (PFS: *p* = 0.610 and OS: *p* = 0.984; Figure 3E,F).

### 3.3 Univariate and multivariate analyses (before PSM)

#### 3.3.1 Progression‐free survival

In univariate analysis, gender, N stage, liver metastasis, chemotherapy regimen, and prior antibiotic use were all found to be related to worse patient PFS, while in multivariate analysis, gender [hazard ratio (HR), 0.817; 95% confidence interval (CI), 0.724–0.923; *p* = 0.001], N stage [hazard ratio (HR), 1.081; 95% CI, 1.017–1.148; *p* = 0.012], liver metastasis (HR, 1.200; 95% CI, 1.051–1.370; *p* = 0.007), and Abx administration (HR, 1.925; 95% CI, 1.717–2.158; *p* < 0.001) were all identified as independent predictors of patient PFS (Table 2).

**TABLE 2:** Univariate and multivariate analyses of PFS and OS among patients with advanced NSCLC receiving chemotherapy before propensity score matching

Variable	Univariate cox regression		Multivariate cox regression	
	HR (95% CI)	*p*‐value	HR (95% CI)	*p*‐value
***Progression‐free survival***				
Age, years				
<60 vs. ≥60	0.955 (0.859–1.061)	0.390		
Gender				
Female vs. Male	0.840 (0.746–0.946)	0.004	0.817 (0.724–0.923)	0.001
T stage				
1 vs.2 vs. 3 vs. 4 vs. 5	1.018 (0.969–1.070)	0.478		
N stage				
0 vs. 1 vs. 2 vs. 3	1.091 (1.028–1.158)	0.004	1.081 (1.017–1.148)	0.012
Histology				
Squamous vs. Non‐Squamous	1.096(0.961–1.251)	0.170		
Hemoglobin, g/L				
>120 vs. ≤120	1.028 (0.919–1.147)	0.648		
Albumin, g/L				
≤40 vs. >40	0.936 (0.831–1.055)	0.277		
Liver metastases				
Yes vs. No	1.204 (1.055–1.373)	0.006	1.200 (1.051–1.370)	0.007
Brain metastases				
Yes vs. No	1.101 (0.971–1.248)	0.132		
Platinum‐based Chemotherapy regimen				
PEM vs. DOC vs. GEM vs. TAX vs. Other	1.046 (1.016–1.077)	0.002	1.020 (0.990–1.050)	0.203
Antiangiogenic therapies				
Yes vs. No	1.090 (0.925–1.295)	0.303	‐	‐
Antibiotic				
Yes vs. No	1.912 (1.707–2.142)	<0.001	1.925 (1.717–2.158)	<0.001
***Overall survival***				
Age, years				
<60 vs. ≥60	0.931 (0.838–1.034)	0.181		
Gender				
Female vs. Male	0.786 (0.699–0.885)	<0.001	0.796 (0.705–0.898)	<0.001
T stage				
1 vs.2 vs. 3 vs. 4 vs. 5	1.051 (0.999–1.106)	0.053		
N stage				
0 vs. 1 vs. 2 vs. 3	1.024 (0.964–1.088)	0.437		
Histology				
Squamous vs. Non‐Squamous	0.816 (0.716–0.930)	0.002	0.869 (0.760–0.993)	0.039
Hemoglobin, g/L				
>120 vs. ≤120	0.992 (0.889–1.108)	0.893		
Albumin, g/L				
≤40 vs. >40	1.080 (0.959–1.215)	0.206		
Liver metastases				
Yes vs. No	1.046 (0.917–1.193)	0.503		
Brain metastases				
Yes vs. No	1.103 (0.973–1.249)	0.125		
Platinum‐based Chemotherapy regimen				
PEM vs. DOC vs. GEM vs. TAX vs. Other	1.010 (0.981–1.040)	0.505		
Antiangiogenic therapies				
Yes vs. No	1.070 (0.907–1.262)	0.423	‐	‐
Antibiotic				
Yes vs. No	1.443 (1.290–1.615)	<0.001	1.452 (1.298–1.625)	<0.001

#### 3.3.2 Overall survival

In univariate analysis, gender, histology, and antibiotic use were all related to shorter patient OS, while in a multivariate analysis, gender (HR, 0.796; 95% CI, 0.705–0.898; *p* < 0.001), histology (HR, 0.869; 95% CI, 0.760–0.993; *p* = 0.039), and Abx administration (HR, 1.452; 95% CI, 1.298–1.625; *p* < 0.001) were all identified as independent predictors of patient OS (Table 2).

### 3.4 Univariate and multivariate analyses (after PSM)

#### 3.4.1 Progression‐free survival

In univariate analysis, gender, N stage, histology, and prior antibiotic use were all found to be related to worse patient PFS, while in multivariate analyses, gender [hazard ratio (HR), 0.761; 95%CI, 0.657–0.882; *p* < 0.001], N stage [hazard ratio (HR), 1.098; 95% CI, 1.015–1.187; *p* = 0.019], histology (HR, 1.330; 95% CI, 1.125–1.573; *p* = 0.001), and Abx administration (HR, 1.865; 95% CI, 1.635–2.126; *p* < 0.001) were all identified as independent predictors of patient PFS (Table 3).

**TABLE 3:** Univariate and multivariate analyses of PFS and OS among patients with advanced NSCLC receiving chemotherapy after 1:1 matching of propensity score matching

Variable	Univariate cox regression		Multivariate cox regression	
	HR (95% CI)	*p*‐value	HR (95% CI)	*p*‐value
***Progression‐free survival***				
Age, years				
<60 vs. ≥60	1.031 (0.905–1.174)	0.651		
Gender				
Female vs. Male	0.819 (0.711–0.944)	0.006	0.761 (0.657–0.882)	<0.001
T stage				
1 vs.2 vs. 3 vs. 4 vs. 5	1.016 (0.956–1.080)	0.609		
N stage				
0 vs. 1 vs. 2 vs. 3	1.091 (1.010–1.178)	0.027	1.098 (1.015–1.187)	0.019
Histology				
Squamous vs. Non‐Squamous	1.180 (1.003–1.389)	0.046	1.330 (1.125–1.573)	0.001
Hemoglobin, g/L				
>120 vs. ≤120	1.032 (0.900–1.183)	0.651		
Albumin, g/L				
≤40 vs. >40	0.912 (0.789–1.054)	0.214		
Liver metastases				
Yes vs. No	1.149 (0.980–1.346)	0.086		
Brain metastases				
Yes vs. No	1.133 (0.962–1.335)	0.134		
Platinum‐based Chemotherapy regimen				
PEM vs. DOC vs. GEM vs. TAX vs. Other	1.021 (0.986–1.058)	0.247		
Antiangiogenic therapies				
Yes vs. No	1.154 (0.957–1.392)	0.134	‐	‐
Antibiotic				
Yes vs. No	1.810 (1.589–2.063)	<0.001	1.865 (1.635–2.126)	<0.001
***Overall survival***				
Age, years				
<60 vs. ≥60	1.006 (0.884–1.145)	0.930		
Gender				
Female vs. Male	0.800 (0.694–0.921)	0.002	0.808 (0.699–0.935)	0.004
T stage				
1 vs.2 vs. 3 vs. 4 vs. 5	1.069 (1.003–1.138)	0.039	1.061 (0.996–1.131)	0.068
N stage				
0 vs. 1 vs. 2 vs. 3	1.014 (0.929–1.096)	0.717		
Histology				
Squamous vs. Non‐Squamous	0.857 (0.729–1.007)	0.060		
Hemoglobin, g/L				
>120 vs. ≤120	1.012 (0.883–1.159)	0.868		
Albumin, g/L				
≤40 vs. >40	1.120 (0.970–1.293)	0.123		
Liver metastases				
Yes vs. No	1.076 (0.918–1.261)	0.367		
Brain metastases				
Yes vs. No	1.128 (0.958–1.328)	0.148		
Platinum‐based Chemotherapy regimen				
PEM vs. DOC vs. GEM vs. TAX vs. Other	1.009 (0.973–1.045)	0.638		
Antiangiogenic therapies				
Yes vs. No	1.030 (0.854–1.244)	0.755	‐	‐
Antibiotic				
Yes vs. No	1.464 (1.285–1.667)	<0.001	1.468 (1.289–1.672)	<0.001

#### 3.4.2 Overall survival

In univariate analysis, gender, T stage, and antibiotic use were all related to shorter patient OS, while in a multivariate analysis, gender (HR, 0.808; 95% CI, 0.699–0.935; *p* = 0.004) and Abx administration (HR, 1.468; 95% CI, 1.289–1.672; *p* < 0.001) were all identified as independent predictors of patient OS (Table 3).

**FIGURE 4:**
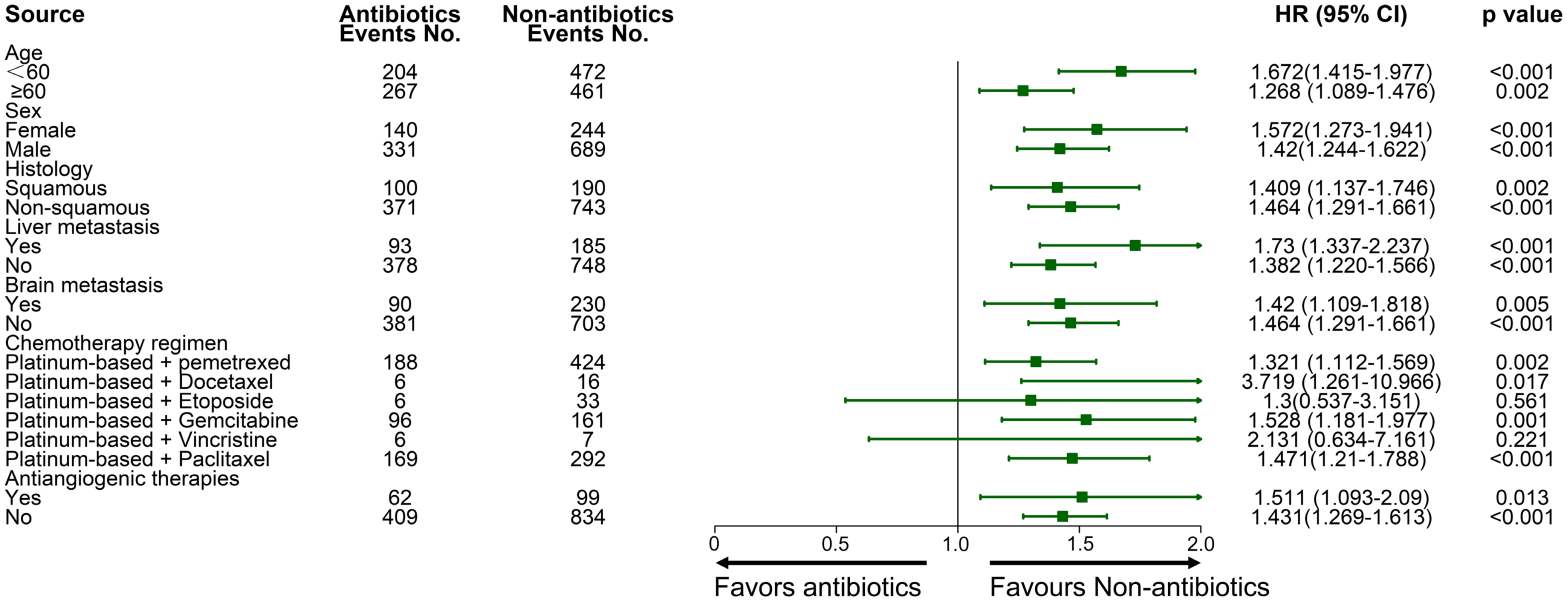
Forest plot for overall survivalThe point estimate of HR = 1 was used as the futility line, the left side of the futility line was the antibiotic treatment group, and the right side of the futility line was the antibiotic untreated group. When the 95% CI of HR included 1, that is, when the horizontal line segment in the forest plot intersected the futility line, it indicated that the difference in survival between the two groups was not statistically significant. When the horizontal line segment did not intersect with the futility line and was to the right of the futility line, it meant that the survival of the group not receiving antibiotics was better

The association between Abx use and patient outcomes was additionally assessed in different patient subgroups. Forest plots revealed that HRs in all subgroups were greater than 1, indicating that Abx use was an unfavorable prognostic factor in all cases (Figure 4). These results were significant in all subgroups other than the platinum‐based + etoposide and platinum‐based + vinorelbine subgroups (*p* < 0.05).

We additionally explored the relationship between antibiotic use duration and patient prognosis, revealing that prolonged antibiotic use was negatively associated with patient PFS (HR, 1.030; 95% CI, 1.007–1.055; *p* = 0.009) and OS (HR, 1.036; 95% CI, 1.014–1.057; *p* = 0.001).

### 3.5 Assessment of the optimal interval between prior Abx treatment and chemotherapy

**FIGURE 5:**
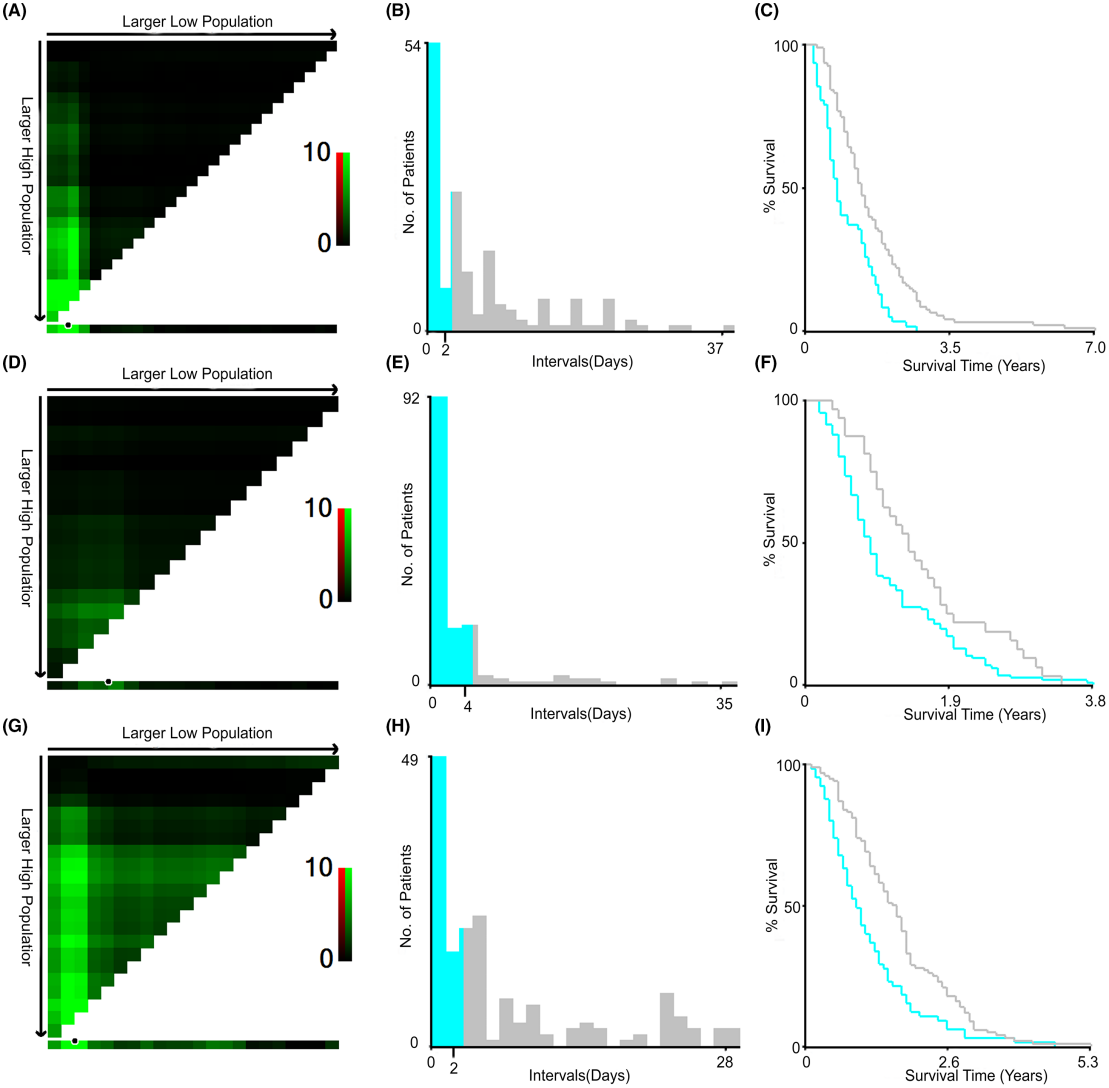
X‐tile analysis of overall survival according to the intervals. (A–C) Optimal interval for patients receiving fluoroquinolones, (D–F). Optimal interval for patients receiving β‐lactamase inhibitor, (G–I). Optimal interval for patients receiving Cephalosporins. X‐tile plots are shown in the left panels, and the optimal cutoff values highlighted by the black circles in left panels are shown in histograms of the entire cohort (middle panels), and Kaplan–Meier plots are displayed in right panels

The interval between the end of antibiotic treatment and the initiation of chemotherapy was additionally assessed, revealing a significant correlation between this interval and patient prognosis. The optimal cutoff values for interval times in patients treated with cephalosporins, β‐lactamase inhibitors, and fluoroquinolone antibiotics were 2, 4, and 2 days, respectively, as calculated using the X‐tile program (Figure 5).

### 3.6 Nomogram construction and validation

**FIGURE 6:**
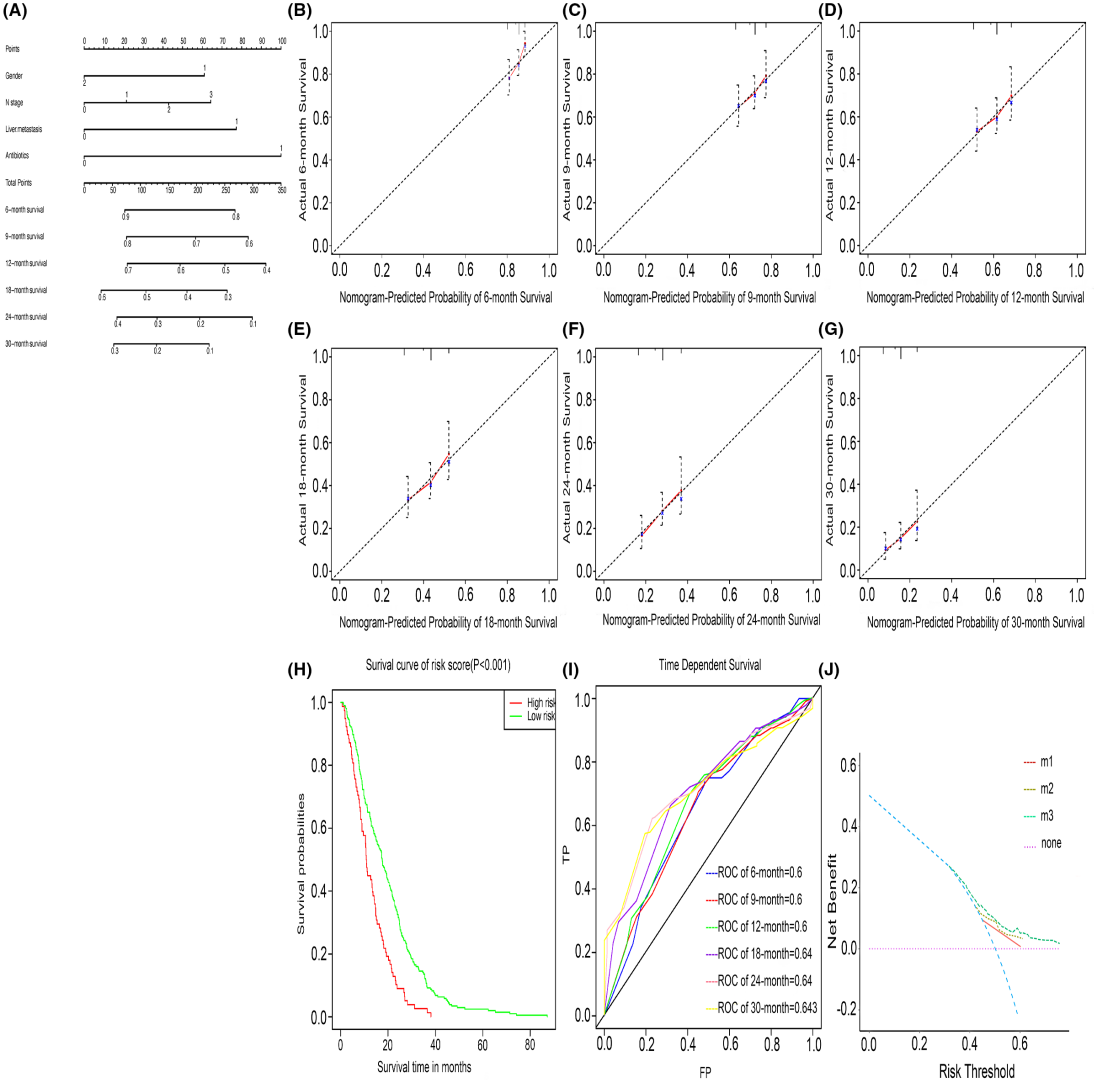
Nomogram construction and validation(A**)** The nomogram assessing 6‐, 9‐, 12‐, 18‐, 24‐, and 30‐month survival in advanced NSCLC receiving chemotherapy. (B–G) Calibration curve for internal validation of the nomogram. The closer the slope of the calibration curve is to 1, the better the predictive ability of the model. (H) Receiver operating curve (ROC). (I) Kaplan–Meier survival curve based on risk stratification system. (J) Decision Curve Analysis of Nomogram. m1: Model that only included antibiotic use; m2: Model established based on gender, N stage, and liver metastasis; m3: Model were established based on all statistically significant variables, including gender, N stage, liver metastases, and antibiotic use. All: The all line means that everyone has a bad survival outcome; None: The none line means that no one has a bad survival outcome. The decision curve is above the all and none lines, indicating that the model has clinical utility

We constructed a novel nomogram model based on the results of multivariate analysis to estimate the 6‐, 9‐, 12‐, 18‐, 24‐, and 30‐month survival rates of patients (Figure 6). The calibration curve showed good consistency between predicted and actual survival. The AUC values of survival in 6‐, 9‐, 12‐, 18‐, 24‐, and 30‐month were 0.6, 0.6, 0.6, 0.64, 0.64, and 0.643, respectively. Patients were divided into high‐risk and low‐risk groups based on their individual risk scores. The Kaplan–Meier curve showed that the survival time of the high‐risk group was shorter than that of the low‐risk group (*p* < 0.001). DCA showed that the model had good survival prediction ability.

Next, we analyzed the impact of antibiotics on patients with advanced NSCLC receiving first‐line immunotherapy and found that OS (15.9 vs. 22.2 months, *p* < 0.001) and PFS (5.0 vs. 9.8 months, *p* < 0.001) were significantly worse in the antibiotic‐treated group than in the antibiotic‐untreated group. Also, prior antibiotic use was shown to be an unfavorable independent prognostic factor for patients (PFS: HR, 5.460; 95% CI, 4.016–7.423; *p* < 0.001; OS: HR, 1.660; 95% CI, 1.255–2.196; *p* < 0.001).

## 4 DISCUSSION

In clinical settings, many NSCLC patients undergo antibiotic treatment prior to the initiation of chemotherapy. Prior preclinical studies shave suggested that antibiotic use can impact gut microbiome diversity and richness, thereby altering the efficacy of different antitumor treatments. Consistently, several studies have reported that antibiotic use can affect the efficacy of immunotherapeutic treatment regimens. Whether antibiotics similarly impact chemotherapy efficacy, however, remains to be established. This study is the first to our knowledge to have compared the effects of different antibiotic treatments on patient prognosis and to define the optimal interval between Abx treatment and chemotherapy initiation.

A growing body of evidence suggests that antibiotic use is associated with poorer survival outcomes in individuals undergoing immune checkpoint inhibitor (ICI) therapy.^8^, ^9^, ^13^ Mechanistically, antibiotics have been found to induce intestinal dysbiosis and to thereby impact immunotherapy efficacy.^17^, ^18^, ^19^ Consistent with findings from trials assessing NSCLC patient outcomes following ICI treatment, we herein determined that the OS and PFS of patients undergoing Abx treatment prior to first‐line chemotherapy were worse than those of patients that did not undergo such treatment, with Abx treatment being an independent predictor of patient prognosis. An intact gut microbiome is essential for systemic chemotherapy in addition to being a prerequisite for immunotherapeutic treatment, and antibiotic use can disrupt the efficacy of several chemotherapeutic regimens.^5^, ^6^, ^20^ Recent reviews have outlined a variety of mechanisms whereby antibiotics can impact chemotherapeutic drug toxicity, including immune regulation, enzymatic degradation, microbial translocation, and decreased microbial diversity.^21^ Ida et al. further demonstrated that gut microbes can modulate immune responses to chemotherapy treatment, with antibiotic administration significantly reducing murine survival time.^6^ However, a separate retrospective analysis revealed that antibiotics had no impact on prognostic outcomes in patients undergoing treatment with immunotherapy plus chemotherapy,^9^ since that study included just five patients in the Abx treatment group. The OAK and POLAR trials similarly determined that Abx use was not associated with PFS or OS among patients undergoing docetaxel treatment.^12^ In the report, researchers assessed antibiotic use within 30 days after chemotherapy initiation, which has previously been demonstrated not to impact patient treatment outcomes.^8^ The study also focused on the second‐line treatment of advanced NSCLC patients, in contrast to the present study. In addition, other report suggested that Abx can impair cisplatin functionality by upregulating VEGFA expression and downregulating CDKN1B and Bax,^22^ potentially contributing to the observed inconsistencies between the prior study and our study.

To improve the convincingness of the results and to visualize the results, we built a nomogram model using statistically significant factors in multivariate analysis. Calibration curves, risk stratification systems, ROC curves, and DCA all showed that the model had strong predictive ability.

Herein, we also verified that antibiotic use affected the efficacy of immunotherapy and observed the negative effects of Abx treatment on survival outcomes for patients undergoing chemotherapeutic treatment plus antiangiogenesis treatment. We also assessed outcomes associated with Abx administration in different subgroups and consistently found that these antibiotics were linked to worse OS irrespective of treatment subgroup, with no significant differences in outcomes between the platinum‐based + etoposide and platinum‐based + vinorelbine treatment subgroups, although these subgroups were both small, limiting the power of statistical comparisons between them.

Intriguingly, we found that different antibiotic regimens were associated with different survival outcomes in individuals undergoing first‐line chemotherapy, consistent with previous ICI‐related reports.^23^, ^24^, ^25^ For example, one analysis of the impact of broad‐spectrum antibiotics on nivolumab efficacy found β‐lactamase inhibitors to have the greatest impact on patient OS and PFS.^26^ In another report, individuals undergoing fluoroquinolone antibiotic treatment exhibited a worse prognosis than individuals being treated with other broad‐spectrum antibiotics.^24^ In our study, we found that *β*‐lactamase inhibitors and fluoroquinolones were associated with a more pronounced difference in survival outcomes among individuals undergoing chemotherapy as compared to cephalosporin treatment. And the clinical outcome of patients receiving β‐lactamase inhibitor antibiotics was the shortest among such NSCLC patients.

We additionally found that the duration of antibiotic treatment was associated with NSCLC patient prognosis, in line with prior reports.^27^, ^28^, ^29^ In a separate study, a shorter Abx treatment duration (6 days) was found to have no impact on patient prognosis relative to a longer treatment course (9 days).^30^ We thus hypothesized that the gut microbiome may impact ICI or chemotherapy efficacy based on complex immunological mechanisms that may recover when a particular “balance point” is reached.

After the discontinuation of antibiotic treatment, the gut microbiome can repopulate, regaining its normal immunoregulatory activity. As such, the interval between Abx treatment and anticancer treatment may be clinically relevant. Multiple retrospective studies have demonstrated that the prognosis of individuals undergoing Abx treatment within 2 months prior to immunotherapy was better than that of patients undergoing Abx treatment within 1 month prior to immunotherapy.^13^, ^14^ In addition, that study found that Abx treatment 3 months prior to the initiation of immunotherapy was not associated with any difference in patient prognosis. Therefore, it is reasonable to assume that an appropriate delay in chemotherapy after discontinuation of antibiotic therapy may benefit the patient's prognosis. However, delaying anticancer treatment in a clinical setting can increase the risk of tumor progression, and it is thus not feasible to delay such treatment while the gut microbiota is fully restored. We thus explored the optimal interval between antibiotic and chemotherapeutic treatment in advanced NSCLC patients, and found that for patients being treated with fluoroquinolone or cephalosporin antibiotics, initial chemotherapy should be postponed for a minimum of 2 days following antibiotic administration, while treatment in patients receiving β‐lactamase inhibitors should be postponed for at least 4 days. While prior work suggests that the gut microbiome cannot return to pre‐antibiotic treatment in that time frame,^31^, ^32^, ^33^ this microbial compartment is substantially affected by diet, region, and environment.^34^ As these prior studies were not conducted in Asia, this may have contributed to differences between their findings and those of the present report owing to genetic or regional variations. In addition, the recovery rate of the microbiome may be nonlinear,^35^ recovering rapidly during the first few days following the discontinuation of antibiotic treatment such that even after a few days, a “balance point” may be reached despite the recovery process being incomplete. Moreover, different bacteria recover at different rates,^36^ with some recovering to pretreatment levels relatively quickly after the cessation of antibiotic treatment. At present, however, these hypotheses remain to be validated in prospective studies examining the process whereby the gut microbiota recovers following different types of antibiotic treatments in an effort to guide precision therapeutic intervention.

Some may argue that establishing a causal relationship between antibiotic use and the prognosis of patients receiving chemotherapy is challenging. First of all, the reason for antibiotic use may have a significant impact on prognosis, in addition, those patients who require long courses of antibiotics or intravenous antibiotics may suffer from more severe infections, which may also potentially have a negative impact on patient prognosis. However, all patients in our study had a PS score of 0 or 1 and were considered to be able to receive platinum‐based chemotherapy. Moreover, there were no significant differences in anticancer treatment regimens between the antibiotic‐treated and antibiotic‐untreated groups. In addition, no infection‐related deaths occurred in any of the included patients, excluding confounding effects of toxic effects. Further, previous studies demonstrated that prior antibiotic therapy affected patient prognosis, whereas antibiotic use during antineoplastic therapy did not affect patient prognosis,^8^ which may imply that infection comorbidity may not be a major determinant of patient prognosis.

There are multiple limitations to the present study. For one, this study was retrospective in nature and thus susceptible to selection bias. In addition, two measured variables differed significantly between the Abx‐treated and Abx‐untreated patient cohorts, potentially confounding our survival analyses. Subgroup analyses were performed in an effort to compensate for this effect. Third, we did not assess the effects of other drugs administered at baseline (PPIs, aspirin, metformin, etc.) and comorbidities on patient outcomes. However, the PS scores of patients included in this study were 0–1, indicating that these patients were in good condition on average. In addition, the methodology of our study is consistent with that of previous studies, and the findings echo those reported in previous studies.^8^, ^11^ Moreover, gut microbiome recovery following antibiotic treatment is a complex process, and further prospective research is warranted to examine the association between a microbial “balance point” and therapeutic outcomes in different patient populations.

## 5 CONCLUSIONS

Overall, our results suggest that the treatment of advanced NSCLC patients with antibiotics prior to the initiation of first‐line chemotherapy was associated with a worse patient prognosis, with these effects being most pronounced for β‐lactamase inhibitors or prolonged treatment duration. Postponing chemotherapeutic initiation in patients undergoing antibiotic treatment may thus incur survival benefits for these patients. These data serve as a reminder that antibiotics should only be administered to patients in the context of strict indications, particularly in oncology‐related clinical settings. However, additional prospective research will be essential to validate these findings.

## AUTHOR CONTRIBUTIONS

Guarantor of integrity of the entire study: **Youling Gong**, Study concepts and design: **Youling Gong**, Literature research: **Youling Gong and Ting Mei**, Clinical studies: **Xiaoman Tian, Ting Mei, and Rui Ao**, Data analysis: **Ting Mei and Youling Gong**, Statistical analysis: **Ting Mei and Youling Gong**, Manuscript preparation: **Youling Gong and Xiaoman**.

## CONFLICT OF INTEREST

The authors report no conflict of interest in this work.

## ETHICAL APPROVAL STATEMENT

This study was approved by the ethics review committee of West China Hospital, Sichuan University, which waived informed consent.

## DECLARATION OF INTEREST

None.

## Data Availability

The data that support the findings of this study are available from the corresponding author upon reasonable request.

## References

[1] Bray F , Ferlay J , Soerjomataram I , Siegel RL , Torre LA , Jemal A . Global cancer statistics 2018: GLOBOCAN estimates of incidence and mortality worldwide for 36 cancers in 185 countries. CA Cancer J Clin. 2018;68(6):394‐424. doi:10.3322/caac.21492 30207593

[2] Rossi A , Di Maio M . Platinum‐based chemotherapy in advanced non‐small‐cell lung cancer: optimal number of treatment cycles. Expert Rev Anticancer Ther. 2016;16(6):653‐660. doi:10.1586/14737140.2016.1170596 27010977

[3] Lange K , Buerger M , Stallmach A , Bruns T . Effects of antibiotics on gut microbiota. Dig Dis. 2016;34(3):260‐268. doi:10.1159/000443360 27028893

[4] Liu J , Liu C , Yue J . Radiotherapy and the gut microbiome: facts and fiction. Radiat Oncol. 2021;16(1):9. doi:10.1186/s13014-020-01735-9 33436010 PMC7805150

[5] Viaud S , Saccheri F , Mignot G , et al. The intestinal microbiota modulates the anticancer immune effects of cyclophosphamide. Science. 2013;342(6161):971‐976. doi:10.1126/science.1240537 24264990 PMC4048947

[6] Iida N , Dzutsev A , Stewart CA , et al. Commensal bacteria control cancer response to therapy by modulating the tumor microenvironment. Science. 2013;342(6161):967‐970. doi:10.1126/science.1240527 24264989 PMC6709532

[7] Hekmatshoar Y , Rahbar Saadat Y , Hosseiniyan Khatibi SM , et al. The impact of tumor and gut microbiotas on cancer therapy: beneficial or detrimental? Life Sci. 2019;233:116680. doi:10.1016/j.lfs.2019.116680 31344431

[8] Pinato DJ , Howlett S , Ottaviani D , et al. Association of Prior Antibiotic Treatment with Survival and Response to immune checkpoint inhibitor therapy in patients with cancer. JAMA Oncol. 2019;5(12):1774‐1778. doi:10.1001/jamaoncol.2019.2785 31513236 PMC6743060

[9] Zhao S , Gao G , Li W , et al. Antibiotics are associated with attenuated efficacy of anti‐PD‐1/PD‐L1 therapies in Chinese patients with advanced non‐small cell lung cancer. Lung Cancer. 2019;130:10‐17. doi:10.1016/j.lungcan.2019.01.017 30885328

[10] Cortellini A , Di Maio M , Nigro O , et al. Differential influence of antibiotic therapy and other medications on oncological outcomes of patients with non‐small cell lung cancer treated with first‐line pembrolizumab versus cytotoxic chemotherapy. J Immunother Cancer. 2021;9(4):e002421. doi:10.1136/jitc-2021-002421 33827906 PMC8031700

[11] Nenclares P , Bhide SA , Sandoval‐Insausti H , et al. Impact of antibiotic use during curative treatment of locally advanced head and neck cancers with chemotherapy and radiotherapy. Eur J Cancer. 2020;131:9‐15. doi:10.1016/j.ejca.2020.02.047 32248073

[12] Chalabi M , Cardona A , Nagarkar DR , et al. Efficacy of chemotherapy and atezolizumab in patients with non‐small‐cell lung cancer receiving antibiotics and proton pump inhibitors: pooled post hoc analyses of the OAK and POPLAR trials. Ann Oncol. 2020;31(4):525‐531. doi:10.1016/j.annonc.2020.01.006 32115349

[13] Derosa L , Hellmann MD , Spaziano M , et al. Negative association of antibiotics on clinical activity of immune checkpoint inhibitors in patients with advanced renal cell and non‐small‐cell lung cancer. Ann Oncol. 2018;29(6):1437‐1444. doi:10.1093/annonc/mdy103 29617710 PMC6354674

[14] Kaderbhai C , Richard C , Fumet JD , et al. Antibiotic use does not appear to influence response to nivolumab. Anticancer Res. 2017;37(6):3195‐3200. 28551664 10.21873/anticanres.11680

[15] Mei T , Yang X , Yu Y , et al. Secondary infections after diagnosis of severe radiation pneumonitis (SRP) among patients with non‐small cell lung cancer: pathogen distributions, choice of empirical antibiotics, and the value of empirical antifungal treatment. Int J Radiat Oncol Biol Phys. 2022;112(1):179‐187. doi:10.1016/j.ijrobp.2021.08.022 34418467

[16] Camp RL , Dolled‐Filhart M , Rimm DL . X‐tile: a new bio‐informatics tool for biomarker assessment and outcome‐based cut‐point optimization. Clin Cancer Res. 2004;10(21):7252‐7259. 15534099 10.1158/1078-0432.CCR-04-0713

[17] Naqash AR , Kihn‐Alarcón AJ , Stavraka C , et al. The role of gut microbiome in modulating response to immune checkpoint inhibitor therapy in cancer. Ann Transl Med. 2021;9(12):1034. doi:10.21037/atm-20-6427 34277834 PMC8267312

[18] Gopalakrishnan V , Spencer CN , Nezi L , et al. Gut microbiome modulates response to anti‐PD‐1 immunotherapy in melanoma patients. Science. 2018;359(6371):97‐103. doi:10.1126/science.aan4236 29097493 PMC5827966

[19] Matson V , Fessler J , Bao R , et al. The commensal microbiome is associated with anti‐PD‐1 efficacy in metastatic melanoma patients. Science. 2018;359(6371):104‐108. doi:10.1126/science.aao3290 29302014 PMC6707353

[20] Ubeda C , Pamer EG . Antibiotics, microbiota, and immune defense. Trends Immunol. 2012;33(9):459‐466. doi:10.1016/j.it.2012.05.003 22677185 PMC3427468

[21] Alexander JL , Wilson ID , Teare J , Marchesi JR , Nicholson JK , Kinross JM . Gut microbiota modulation of chemotherapy efficacy and toxicity. Nat Rev Gastroenterol Hepatol. 2017;14(6):356‐365. doi:10.1038/nrgastro.2017.20 28270698

[22] Gui QF , Lu HF , Zhang CX , Xu ZR , Yang YH . Well‐balanced commensal microbiota contributes to anti‐cancer response in a lung cancer mouse model. Genet Mol Res. 2015;14(2):5642‐5651. doi:10.4238/2015.May.25.16 26125762

[23] Zimmermann P , Curtis N . The effect of antibiotics on the composition of the intestinal microbiota ‐ a systematic review. J Infect. 2019;79(6):471‐489. doi:10.1016/j.jinf.2019.10.008 31629863

[24] Lu P‐H , Tsai T‐C , Chang JW‐C , Deng S‐T , Cheng C‐Y . Association of prior fluoroquinolone treatment with survival outcomes of immune checkpoint inhibitors in Asia. J Clin Pharm Ther. 2021;46(2):408‐414. doi:10.1111/jcpt.13298 33332621

[25] Iizumi T , Battaglia T , Ruiz V , Perez Perez GI . Gut microbiome and antibiotics. Arch Med Res. 2017;48(8):727‐734. doi:10.1016/j.arcmed.2017.11.004 29221800

[26] Geum MJ , Kim C , Kang JE , et al. Broad‐Spectrum antibiotic regimen affects survival in patients receiving nivolumab for non‐small cell lung cancer. Pharmaceuticals (Basel). 2021;14(5):445. doi:10.3390/ph14050445 34066877 PMC8151442

[27] Galli G , Triulzi T , Proto C , et al. Association between antibiotic‐immunotherapy exposure ratio and outcome in metastatic non small cell lung cancer. Lung Cancer. 2019;132:72‐78. doi:10.1016/j.lungcan.2019.04.008 31097097

[28] Iglesias‐Santamaría A . Impact of antibiotic use and other concomitant medications on the efficacy of immune checkpoint inhibitors in patients with advanced cancer. Clin Transl Oncol. 2020;22(9):1481‐1490. doi:10.1007/s12094-019-02282-w 31919759

[29] Tinsley N , Zhou C , Tan G , et al. Cumulative antibiotic use significantly decreases efficacy of checkpoint inhibitors in patients with advanced cancer. Oncologist. 2020;25(1):55‐63. doi:10.1634/theoncologist.2019-0160 31292268 PMC6964118

[30] Elkrief A , Derosa L , Kroemer G , Zitvogel L , Routy B . The negative impact of antibiotics on outcomes in cancer patients treated with immunotherapy: a new independent prognostic factor? Ann Oncol. 2019;30(10):1572‐1579. doi:10.1093/annonc/mdz206 31268133

[31] Dethlefsen L , Relman DA . Incomplete recovery and individualized responses of the human distal gut microbiota to repeated antibiotic perturbation. Proc Natl Acad Sci U S A. 2011;108(Suppl 1):4554‐4561. doi:10.1073/pnas.1000087107 20847294 PMC3063582

[32] Brismar B , Edlund C , Nord CE . Impact of cefpodoxime proxetil and amoxicillin on the normal oral and intestinal microflora. Eur J Clin Microbiol Infect Dis. 1993;12(9):714‐719. 8243490 10.1007/BF02009388

[33] Zaura E , Brandt BW , Teixeira de Mattos MJ , et al. Same Exposure but Two Radically Different Responses to Antibiotics: Resilience of the Salivary Microbiome versus Long‐Term Microbial Shifts in Feces. mBio. 2015;6(6):e01693‐e01615. doi:10.1128/mBio.01693-15 26556275 PMC4659469

[34] Ng KM , Aranda‐Díaz A , Tropini C , et al. Recovery of the gut microbiota after antibiotics depends on host diet, community context, and environmental reservoirs. Cell Host Microbe. 2020;28(4):628. doi:10.1016/j.chom.2020.09.001 33031771

[35] Palleja A , Mikkelsen KH , Forslund SK , et al. Recovery of gut microbiota of healthy adults following antibiotic exposure. Nat Microbiol. 2018;3(11):1255‐1265. doi:10.1038/s41564-018-0257-9 30349083

[36] Ramirez J , Guarner F , Bustos Fernandez L , Maruy A , Sdepanian VL , Cohen H . Antibiotics as major disruptors of gut microbiota. Front Cell Infect Microbiol. 2020;10:572912. doi:10.3389/fcimb.2020.572912 33330122 PMC7732679

